# Stellar occultations: a gateway to advances in planetary science

**DOI:** 10.1098/rsta.2024.0202

**Published:** 2025-02-27

**Authors:** D. Souami, R. Sfair, S. Renner, M. El Moutamid

**Affiliations:** ^1^ LIRA, CNRS, Observatoire de Paris, Université PSL, Sorbonne Université, Université Paris Cité, CY Cergy Paris Université, Meudon, 92190, France; ^2^ naXys, Department of Mathematics, University of Namur, Rue de Bruxelles 61, Namur 5000, Belgium; ^3^ São Paulo State University (UNESP), School of Engineering and Sciences, Guaratinguetá 12516-410, Brazil; ^4^ Institute of Astronomy and Astrophysics, University of Tübingen, Auf der Morgenstelle 10, Tübingen 72076, Germany; ^5^ IMCCE, Observatoire de Paris, Université PSL, Sorbonne Université, Université de Lille, CNRS UMR 8028, 77 Avenue Denfert-Rochereau, Paris 75014, France; ^6^ Department of Space Studies, Southwest Research Institute, 1301 Walnut Street, Suite 400, Boulder, CO 80301, USA

**Keywords:** Planetary Science, occultations, Citizen Science, Dynamics

## Introduction

Ground-based stellar occultations are a powerful method to explore the Solar System. The method is elegantly simple yet powerful: it consists of measuring the variation of starlight as a Solar System object passes in front of the star in the plane of the sky. The physical information on the object is inferred from studying the disappearance and reappearance of the star. This provides milli-arcsec (mas) astrometric accuracy on the occulting object, when associated with the ESA/*Gaia* mission stellar catalogues [1–4], thus significantly improving ephemerides. Moreover, occultations offer long-term monitoring of objects that time-limited space missions cannot provide. Withal, this technique is accessible to both amateur and professional astronomers, thereby democratising the opportunity for everyone to engage in ground-breaking discoveries using small telescopes ([5–7], from this special issue).

Good-quality stellar occultation data (i.e. high-rate data acquisition and time accuracy ranging from a few milliseconds to a few tens of microseconds) allow high spatial resolution at the km level in measuring astrometric positions, sizes and shapes for outer Solar System objects, for example. Thus overcoming adaptive optics imaging with large telescopes by orders of magnitude and even surpassing the spatial resolution of space telescopes such as the James Webb Space Telescope (JWST) [8]. Extensive reviews on this powerful method of stellar occultation to study solar system objects can be found in [9] and references therein, as well as [10] from this special issue.

Moreover, stellar occultations are particularly sensitive to tenuous atmospheres, down to nano-bar levels. In this special issue [11], we present a comprehensive review on how stellar occultations enable to probe atmospheric density, pressure, and temperature profiles around typical ranges of a few µbar. Furthermore, temperature profiles from occultations, at 100−0.1 µbar, complement profiles at deeper levels from thermal spectroscopy (including those from JWST), permitting, for instance, investigation of the response of stratospheres to insolation changes.

This special issue on ‘*Major Advances in Planetary Sciences thanks to Stellar Occultations’* emerged from papers that were presented at the ‘Colloquium in honour of Professor Bruno Sicardy’, which was held from 22 April to 26 April 2024 at the Observatoire de Paris, in the historic *Cassini Hall* of the *Bâtiment Perrault* that was built between 1667 and 1672.

The workshop initially planned to celebrate the 40^th^ year anniversary of the discovery of Neptune’s incomplete ring-like structures (i.e. arcs) by occultation, celebrated also the career of Prof. Bruno Sicardy who is one of the co-discoverers of these arcs, and who retired in January 2024.

The papers in this issue explore research directions where stellar occultations have proven pivotal to advancing planetary science. The issue presents review papers (from atmosphere studies [11], discussion on rings’ origins in the Solar System [12], to how occultations by the outer Solar System objects constrain the formation models [10]), papers presenting the outcomes of recent observational data and other that are more theoretical covering the dynamical topics.

In the observational data paper, we have the case of comet 29P/Schwassmann-Wachmann 1 nucleus’s near-environment [13], the Jupiter Trojan (1437) Diomedes [14], probing the non-uniform longitude distribution in Chariklo’s main ring [15] and a review paper on binary trans-neptunian systems, through the example of Vanth and Weywot [16], satellites of the dwarf planets Orcus and Quaoar, respectively. Indeed studying the physical properties of these objects and their distributions provides valuable constraints of the Solar System formation models [10].

The most theoretical papers cover different topics from planetary ring dynamics (such as the global dynamics of Saturn’s arcs [17] and the dust production models that would feed the G-ring arc [18]), the modelling of internal structures [19] of asteroids, to the modelling of non-gravitational (Yarkovsky and the Yarkovsky–O’Keefe–Radzievskii–Paddack - YORP -) effects [20] in the case of the Near-Earth Asteroid (3200) Phæthon, target of the JAXA Destiny+ mission, and which has been observed several times by stellar occultations.

The ProAm (Professional–Amateur) collaborations have always been at the heart of occultations with large international observing campaigns coordinated on all continents. We take advantage of this opportunity to express our gratitude and uttermost respect to our collaborators, Citizen Scientists, across the globe for their time, commitment, dedication and their contributions to planetary science.

The papers for this issue were selected so that they give a comprehensive picture of all the science that is achieved and that can be achieved over the next decades using stellar occultations to probe our Solar System. These nonpareil capabilities of stellar occultation have provided outstanding results over several decades. As is often the case in science, an efficient technique not only improves our current knowledge but also reveals unexpected processes that have far-reaching consequences on our understanding of natural phenomena.

**Figure 1.**
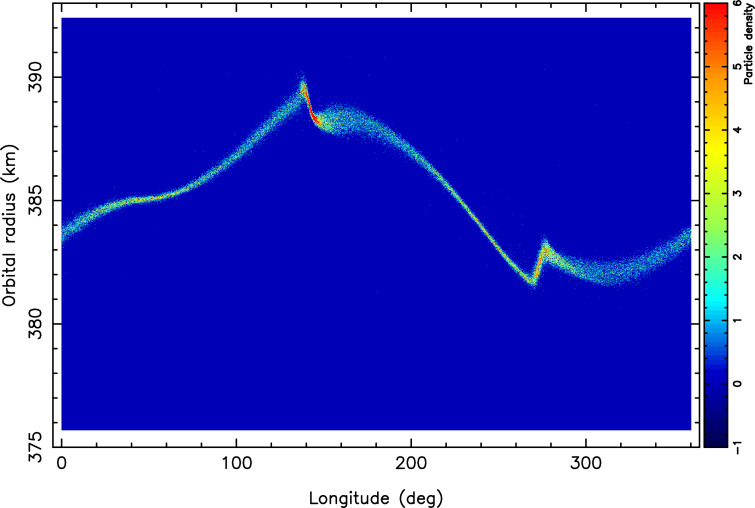
An N-body simulation illustrating the confinement of a ring around the Centaur object Chariklo**.** The simulation is fully three-dimensional and azimuthally complete. It accounts for inelastic collisions (but no gravitational interactions) between 40 000 particles of 25 m radius, which are perturbed by a mass anomaly representing 0.3% of Chariklo’s total mass. The ring particles are initially placed on circular orbits around the 1/3 resonance with the central body, where the particles complete one revolution when Chariklo completes three rotations. The image shows a polar density plot of the ring after 205 years, corresponding to approximately 86 000 particle revolutions. The horizontal axis shows the longitudes of the particles, while the vertical axis shows their distances to Chariklo’s centre, knowing that the 1/3 resonance has been placed at 385 km (the radius of Chariklo’s main ring). After an initial viscous spreading caused by collisions, the 1/3 resonance excites the orbital eccentricities of the particles, leading to the nonlinear forcing of various modes with azimuthal numbers of 1, 2, 3.... These modes cause a reversal of the angular momentum flow through the ring, ultimately, leading to its confinement (credit: Heikki Salo and Bruno Sicardy).

These papers allow us to sketch the frame of the future big questions that can be addressed using stellar occultations for the next decades in planetary science. In particular, the following questions came out of some papers and also the discussions during the colloquium:

- In the context of the expected growing volume of data from the *Gaia* catalogues of Solar System objects (data release 4 expected in mid-2026, data release 5*—the grand finale-* expected in the early 2030s) and the upcoming Large Synoptic Survey Telescope of the Vera C. Rubin Observatory (with first light expected in June 2025), we are expecting the number of trans-neptunian objects to increase as it is expected that thousands of large trans-neptunian objects and Centaurs discovered by the start of 2030. Thus, future exploration of the outer Solar System using stellar occultations holds promising potential for advancing our understanding of distant and poorly explored regions of space. As the sample size of objects increases and as the network of observers expands and technology advances, this technique will yield more frequent and precise observations of objects in the Kuiper Belt, the Oort Cloud and beyond. Characterising these most pristine objects will inform and help improve our theoretical and numerical models for the formation and evolution of the Solar System [10]. This will allow a comprehensive mapping of the outer Solar System providing a better understanding of the Solar System.
- Ring systems: Over the last decade, unexpected rings and arcs encircling a few small bodies of Solar System were discovered. Three unique ring systems have been confirmed, so far: with the first ring discovered in 2013 around the Centaur Chariklo [21], followed by the ring around the dwarf planets Haumea [22] in 2017, and the most recent puzzling discovery (in 2022) of an unexpected inhomogeneous dense ring around Quaoar [23] followed by the discovery (in 2023) of a second ring around this same object [24]. Both rings are too far from the central body, thus questioning the well-established concept of the ‘Roche limit’. The discoveries of these rings open a new set of questions [12] as to their origins, their longevities, the mechanisms governing the confinement of the material, etc.The arcs of Neptune, which were discovered four decades ago, are still challenging our theoretical models, while these new ring systems seem to call into question our current understanding of ring systems. Figure 1 illustrates the complexity of the open questions before us, in the case of an attempt to modelling the dynamics of the confinement of a ring around the Centaur Chariklo.As a consequence of the thousands of trans-neptunian objects and Centaurs that will be discovered by large surveys, by the end of the decade, many more satellites and ring systems are yet to be discovered and stellar occultations are the only method to achieve that.As of now, a sample of approximately 30 of these remote objects have been extensively probed with stellar occultations, leading to the discovery of three confirmed ring systems. It is safe to say that ‘many more rings are yet to be discovered’.
- In the aforementioned context of growing data volume and a huge increase in the number of targets and occultation events, the community at large will have to make adjustments. One of the future challenges will be to develop the proper tools and new protocoles, so that the Citizen Scientists continue to enjoy the scientific challenges without being overwhelmed with large number of observable events and continue to be part of great advances in Planetary Science, and so that the scientific data are efficiently exploited and shared in the community.

Aside from being a very powerful technique, the method of stellar occultations, it is also a human adventure built on international collaborations, exchanges and mutual respect. The community expands beyond the walls of research institutions and beyond borders. The diversity of the paper in this issue is proof of that.

The colloquium was organised by a group of former students and post-docs of Professor Bruno Sicardy, who are also guest editors of this special issue. The participants in this colloquium were either long-term or recent collaborators of Prof. Sicardy, students of former students, or indirect collaborators through his former students. Part of the extended Sicardy’s scientific family gathered in this historical setting, with a hybrid component for the meeting to accommodate those who could not travel.

The guest editors are honoured to be able to extend this historical setting by publishing this special issue on ‘*Major Advances in Planetary Sciences thanks to Stellar Occultations*’ in *Philosophical Transactions of the Royal Society A*, which is celebrating its 360 year anniversary in 2025 and which has published the works of Mr. (Sir) Isaac Newton (1642–1727).

**Figure 2.**
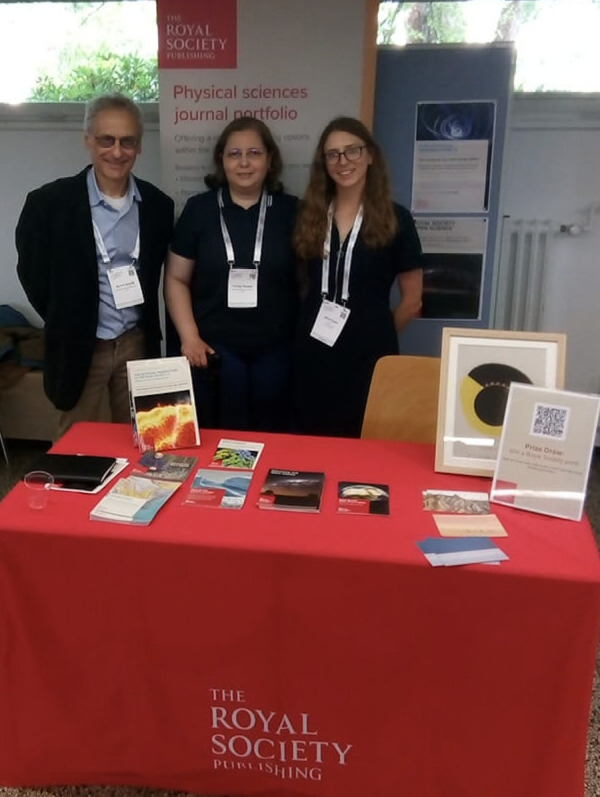
Photograph taken at the Europlanet Science Congress, September 2024.

As a final note to the human adventure, we publish here a photograph taken at the Europlanet Science Congress figure 2 that was held in Berlin, in September 2024. After months of exchanging emails, we met in person (from left to right on the figure) Bruno Sicardy, Damya Souami, and Alice Power (Commissioning Editor at *Philosophical Transactions A*).

## Data Availability

This article has no additional data.
